# A Novel Effective Nanoadjuvant System for Poultry Vaccines

**DOI:** 10.3390/vaccines14070613

**Published:** 2026-07-14

**Authors:** Fakry F. Mohamed, Hassanein H. Abozeid, Matt Murray, Adel M. Talaat

**Affiliations:** 1 Department of Pathobiological Sciences, School of Veterinary Medicine, University of Wisconsin-Madison, Madison, WI 53706, USA; framadan@wisc.edu (F.F.M.); hassanein.abozeid@wisc.edu (H.H.A.); mmmurray9@wisc.edu (M.M.); 2 Department of Virology, Faculty of Veterinary Medicine, Zagazig University, Zagazig 44511, Sharkia, Egypt; 3 Department of Poultry Diseases, Faculty of Veterinary Medicine, Cairo University, Giza 12211, Egypt; 4 Vireo Vaccine International, Middleton 53562, WI, USA

**Keywords:** nanovaccines, DNA vaccine, infectious bronchitis virus, LNPs, QTAP

## Abstract

**Background**: Nucleic acid vaccines adjuvanted with lipid nanoparticles (LNPs) are a simple and effective means of protection against infectious diseases through the delivery of antigen(s) that elicit(s) specific immunity. DNA vaccines are not commercially available for poultry mainly due to low protective efficacy and the associated production cost. **Methods**: We introduced here a novel nanoadjuvant system (namely, QTAP) comprising DOTAP and Quil-A for efficient delivery of plasmid DNA (pDNA) constructs into cells and boosting immune responses of chickens. Variable QTAP-pDNA-LNPs formulas were investigated for their physicochemical characteristics, cellular transfection, and *in vivo* efficacy in a vaccine/challenge model using pDNA encoding both S and N proteins of avian Infectious Bronchitis Virus (IBV). **Results**: Under electron microscopy, all LNP formulas were spherical in shape and had an overall size range of 45.4–376.3 nm. The formula with the smallest size had the lowest pDNA encapsulation and the highest polydispersity index (PDI). On the contrary, formulas with larger sizes and lower PDI values encapsulated a higher concentration of plasmid payload. Interestingly, the high-payload vaccine formula was stable at both 25 °C and 4 °C for up to 6 weeks of storage, but its gene expression dropped beyond this point at both temperatures. Following immunization of chickens, all groups were challenged with a virulent IBV. Surprisingly, both formulas were able to reduce the IBV viral shedding, indicating their effectiveness even when low concentrations of pDNA were incorporated in LNPs. However, pre-challenge sera from immunized chicks did not elicit IBV-specific antibodies, compared to birds immunized with a commercial live-attenuated IBV vaccine. **Conclusions**: This study suggests that the QTAP nanoadjuvant system is a safe and effective adjuvant for DNA immunization in poultry that could also be further developed for commercialization.

## 1. Introduction

Compared to modified live-attenuated vaccines (MLVs), nucleic acid-based (DNA or mRNA) ones do not retain residual pathogenicity and lack the capacity to revert to virulence [1,2,3]. Both MLVs and nucleic acid platforms efficiently mount robust and vaccine-specific immunity, distinguishing them from less immunogenic inactivated vaccines [1,4,5] or other economically demanding and purification-complex vaccine options [6]. Upon entering host cells, plasmid DNA (pDNA) undergoes cellular processing to ultimately present antigenic peptides on the cell surface via major histocompatibility complexes (MHC) class I and II [7]. This process triggers a broad spectrum of immune reactions, encompassing both humoral antibody- and cellular T-cell-mediated immune responses. Furthermore, DNA vaccines offer the distinct advantages of streamlined production at low manufacturing costs and superior thermal stability compared to their RNA counterparts [8,9]. In poultry, DNA vaccination represents a safe, stable, immunogenic, and DIVA-compatible (Differentiating Infected from Vaccinated Animals) alternative [10,11,12,13]. Crucially, DNA vaccines eliminate the risk of viral shedding associated with MLVs. They are also highly suitable for *in ovo* administration even in the presence of maternally derived antibodies (MDAs) [1,14,15]. Acknowledging their potential, the United States Department of Agriculture (USDA) granted conditional approval to the first chicken DNA vaccine against H5N1 avian influenza virus (AIV) [16]. Nevertheless, the application of poultry DNA vaccination remains constrained, largely due to practical hurdles in identifying protective, immunogenic vaccine constructs and the practical formulation of plasmid vaccines for field application [1]. To address these challenges, the current study seeks to establish an adjuvanted DNA vaccine platform for poultry utilizing a newly designated lipid nanoparticle (LNP) system (termed QTAP) that was further evaluated using the infectious bronchitis virus (IBV) challenge model.

IBV is a major threat to the global poultry industry, causing an acute contagious disease to chickens at all ages by targeting the respiratory, renal, and reproductive systems of birds [4]. The classical Massachusetts IBV-M41 strain is known to cause respiratory distress characterized by dyspnea, coughing, rales, and oculonasal discharge, especially in young chickens [17]. While MLVs are widely implemented to manage IBV outbreaks, concerns persist regarding their reversion to virulence and potential genomic recombination with circulating field strains [4,18]. Furthermore, minor sequence variations (as low as 4%) within the hypervariable regions of the viral surface spike protein (IBV-S) can culminate in complete vaccination failure [19]. The IBV genome encodes structural and non-structural proteins, including IBV-S and nucleocapsid (IBV-N) [20]. Previous studies established that DNA vaccines encoding IBV-S and/or IBV-N can successfully trigger protective immunity in chickens [11,21]. Given the continuous emergence of novel IBV serotypes and genotypes, there is an urgent demand for a safe, highly effective, and rapidly adaptable vaccine platform against IBV, directly aligning with the objectives of this study.

Optimizing DNA vaccines requires a careful consideration of plasmid design, delivery methodologies, formulation strategies, and the integration of adjuvants [15]. To augment vaccine-induced immune responses in birds, plasmid vaccines are commonly co-administered with traditional or molecular adjuvants [22]. In this context, LNPs can function as potent nanoadjuvants. By adsorbing or encapsulating nucleic acids and companion antigens, they enhance structural protection, stability, biodistribution, and cellular uptake of DNA, which subsequently drives robust immune responses [15,23]. Generally, nucleic acid-based vaccines utilizing LNPs have demonstrated high efficacy against various avian viruses [24,25]. Among these formulation components, the cationic lipid DOTAP (1,2-Dioleoyl-3-trimethylammonium-propane) is essential to assemble the negatively charged nucleic acids into stable LNPs [26,27,28]. Because the surface of DOTAP-LNPs remains positively charged at physiological pH, it amplifies both cellular uptake and antigen presentation by immune cells [29,30], allowing DOTAP-LNPs to serve as an effective dual delivery and adjuvant system [31]. Consequently, DOTAP-based formulations are expected to precisely deliver antigens intracellularly while circumventing common bottlenecks such as endosomal escape and nucleic acid degradation [32]. However, because DOTAP may also be cytotoxic, the nitrogen-to-phosphate (N/P) ratio, representing the balance between the positive amine groups of DOTAP and the negative phosphate groups of the nucleic acid backbone, must be meticulously optimized to achieve ideal parameter balances for successful *in vivo* expression and immunogenicity [33,34]. This is largely controlled by the precise concentration of DOTAP utilized [35]. Building upon these principles, we developed the QTAP-pDNA-LNPs vaccine platform by incorporating the saponin Quil-A to further optimize the formulation characteristics and boost host immune responses.

As a prospective approach, this study characterizes distinct Quil-A adjuvanted DOTAP-pDNA-LNPs (QTAP-pDNA-LNPs) vaccine formulations designed for administration in chickens against IBV. The foundational QTAP vaccine technology was previously established as a LNP nanoadjuvant system to deliver *Mycobacterium avium*-encoded antigens, combining DOTAP and Quil-A, to achieve efficient cellular uptake and vaccine-induced immunity [36]. Here, we introduced different modifications in QTAP-pDNA-LNPs vaccine formulations and evaluated their physicochemical characteristics prior to validating the efficacy of two vaccine formulas against an IBV-M41 challenge in specific pathogen free (SPF) and commercial chickens. Ultimately, the engineered QTAP-pDNA-LNPs vaccine formulas were able to meet baseline criteria of nanovaccines in size, encapsulation, and cell transfection, while being able to reduce the IBV viral shedding in challenged birds.

## 2. Materials and Methods

### 2.1. Cells and Virus

Human embryonic kidney (HEK293T) cells from ATCC, Manassas, VA, USA (Accession number CRL-3216) were a kind gift from Osorio’s lab at UW-Madison. Cells were maintained in Dulbecco’s Modified Eagle’s media containing 10% fetal bovine serum and 1% penicillin-streptomycin. The virulent IBV-M41 was propagated in SPF fertile eggs, and the virus stock was retrieved from allantoic fluids on day-3 post-inoculation. The virus stock was titrated in SPF eggs, and its titer was expressed as 50% egg infective dose (EID_50_).

### 2.2. IBV Plasmid DNA Constructs

IBV-S and IBV-N complete coding sequences were synthesized based on the reference IBV-M41 strain. The synthesized DNA was back-translated, chicken-codon-optimized (cco), and cloned between EcoRI and NotI restriction sites into the backbone of pCAG expression plasmid. In addition, the Kozak sequence (CCACC) was inserted upstream of the start codon (ATG). The recombinant plasmids were sequenced at the UW-Madison Biotechnology Center to verify the presence of gene inserts (IBV-S or IBV-N), which was further confirmed by restriction enzyme cutting. The IBV-S sequence contained the multibasic cleavage site (RRFRR ↓ SIT) that is responsible for proteolytic processing of full IBV-S protein into S1 and S2 subunits [37]. Another IBV-S pDNA construct with a deleted cleavage site (dCS; namely locked S) was used as a control.

### 2.3. Synthesis and Characterization of QTAP-pDNA-LNPs

QTAP-pDNA-LNPs were formulated by equal mixing of mixtures-1 and -2 in a dropwise manner while vortexing to allow maximum uniformity of LNPs. Mixture-1 contained Quil-A (13.33 µg final concentration per vaccine dose), 100 µg of pDNA (e.g., Green Fluorescent Protein “GFP” or IBV S + N separate or combined) dissolved in 0.5 M sodium sulfate, and nuclease-free water. Mixture-2 contained the DOTAP (Avanti Research, Alabaster, AL, USA.) that was initially dissolved in a 5% glucose solution (Formula I) or 100% ethanol “EtOH” (Formulas II and III). Dissolved DOTAP was further diluted in 5 mM sodium acetate to reach a final concentration of 0.25 mg/mL, 1.25 mg/mL, and 2.5 mg/mL for Formulas I, II, and III, respectively. The prepared LNPs were incubated at room temperature for 30 min before sonication by Misonix Sonicator^®^ 3000 and Microtip Probe K-04711-89 (Qsonica Sonicators, Newtown, CT, USA) at output power of 3 W and frequency of 20 kHz for 1–3 times (20 s each) with resting intervals on ice.

The physicochemical characteristics of QTAP-pDNA-LNPs formulas, including particle size (d.nm), pDNA encapsulation efficiency, polydispersity index (PDI), zeta potential (mV), and their ability to transfect HEK293T cells, were compared. The LNP particle size was measured by dynamic light scattering (DLS) analysis (Malvern Instrument Ltd., Westborough, MA, USA) and Zetasizer Ver. 7.03 to report the intensity PSD particle sizes (intensity-weighted particle size distribution) at 25 °C. The refractive index (RI) was set to 1.529 for DOTAP with 0.001 adsorption. Milli-Q water (RI of 1.333) was used as a dispersant for intensity PSD sizing. For the zeta potential, particles were dispersed in 1 mM sodium chloride before being measured at an average conductivity of 0.15 ± 0.02 mS/cm. The encapsulation efficiency was measured by detecting the concentration of free DNA in the supernatants of vaccine formulas after precipitation of LNPs by centrifugation (21,000 rpm for 10 min at 4 °C) and by applying the following equation: (concentration of free DNA/concentration of total DNA) × 100. The three formulas were further examined by transmission electron microscopy (TEM) at SMPH Electron Microscope Core of the UW-Madison using the Philips CM120 TEM (FEI, Eindhoven, The Netherlands) at 80 kV. The average size of QTAP-pDNA-LNPs was evaluated by using the ImageJ software Version 1.54p [38].

For the stability studies, the Formula II vaccine dose was prepared and then divided into aliquots, stored at either 37 °C or 4 °C. Aliquots were further tested at the indicated time points (day-0, day-10, day-24, week-6, and week-12) post-formulation by transfection of 1 µg pDNA into HEK293T cells. The same physicochemical parameters were also measured at each time point.

### 2.4. Western Blotting

To evaluate the expression of IBV-S and IBV-N pDNA vaccine constructs, HEK293T cells were seeded overnight in 12-well tissue culture plates until they reached 80–90% confluency. Cells were transfected with 1 µg IBV-S or IBV-N pDNA using the TransIT-LT1 DNA transfection reagent (MiRus, Madison, WI, USA) or one of vaccine formulas, unless otherwise indicated. Three days post-transfection, cells were harvested in PBS, washed, sonicated, and processed with 6x Laemmli buffer (45 μL of 6% Sodium Dodecyl Sulfate, 3 μL of 1% Bromophenol Blue, and 5 μL of 6 M dithiothreitol) at 95 °C for 10 min. Cell lysates were loaded on Mini-PROTEAN^®^ TGX™ Precast Gels (Bio-Rad, Hercules, CA, USA). The gel was dry blotted using the Trans-Blot Turbo Transfer System (Bio-Rad, Hercules, CA, USA). The PVDF membranes were blocked overnight with 5% skim milk at 4 °C, incubated with anti-IBV antibody (25 °C/1 h), washed 3× for 10 min each, incubated with horseradish peroxidase (HRP) conjugated anti-chicken IgY antibody (Novus Biologicals, Centennial, CO, USA) for 25 °C/1 h, washed 3× for 10 min each, and finally incubated with the SuperSignal™ West Femto Maximum Sensitivity Substrate (ThermoFisher Scientific, Waltham, MA, USA) at 25 °C for 5–10 min.

### 2.5. Chicken Vaccination and Challenge

One-day-old leghorn SPF chicks from AVSBio (Charles River Laboratories, Roanoke, IL, USA) or commercial chicks from a local hatchery were used to evaluate the efficacy of Formula I and Formula II study vaccine candidates, respectively. The SPF (*n* = 36) and commercial (*n* = 32) chicks were divided into 4 groups (*n* = 8–9 birds/group). SPF chicken groups received one of the following: PBS (as a negative control), MLV (Poulvac IBV Mass, Zoetis “Charles City, Iowa, USA”, as a positive control), QTAP-IBV-S + N-LNPs Formula I (vaccine candidate encoding both S and N proteins separately), or naked DNA (unencapsulated pDNA-IBV-S + N as a non-adjuvanted control). All birds were vaccinated oculonasally (ON), except for one group in the commercial chicken trial that received the prime dose subcutaneously (SC). For commercial chickens, the previously mentioned last two groups were replaced by the QTAP-IBV-S + N-LNPs Formula II administered SC/ON or ON/ON, respectively. The MLV group in both trials received a single ON dose of attenuated IBV-M41 on day-1 of age, according to the manufacturer’s instructions. While chicks received QTAP-pDNA-LNPs vaccines 2 times (primarily ON/ON and SC/ON in one group) on day-1 and day-14 of age for prime-boost. Both formulas aimed to encapsulate a total of 100 µg pDNA, divided equally on IBV-S (50 µg) and IBV-N (50 µg) pDNA vaccine constructs. On day-28 of age (2 weeks post-booster vaccination), blood (sera) samples were collected from birds before being challenged with IBV-M41 virus at infectious dose of 1 × 10^4^ EID_50_. On day-5 post-challenge (DPC-5), oropharyngeal swabs were collected for RNA extraction and viral load titration using qRT-PCR.

### 2.6. Viral Load Measurement by qRT-PCR

The viral RNA was isolated from collected swabs by using the Direct-Zol RNA MicroPrep kit (Zymo Research, Irvine, CA, USA) according to the manufacturer’s instructions. A one-step qRT-PCR reaction was conducted by using StepOnePlus real-time PCR system (Applied Biosystems, Foster City, CA, USA) to amplify a 143 bp region in the 5’UTR of IBV genome using a specific set of primers and TaqMan probe [39]. The TaqMan Fast Virus 1-Step Master Mix (ThermoFisher Scientific, Waltham, MA, USA) was used in the reaction, which ran under the following conditions: 50 °C/5 min for reverse transcription, 95 °C/20 s for initial denaturation, and 40× cycles of 95 °C/3 s for denaturation and 60 °C/30 s for annealing and extension. A previously titrated IBV-M41 virus was used for the calculation of the standard curve. 

### 2.7. IBV-Specific Indirect ELISA

ELISA was developed to scan the pre-challenge sera for vaccine-induced humoral immune responses against the IBV-M41 virus [11] with modifications. To purify the IBV-M41, the virus suspension (allantoic fluid) was mixed at ratio of 1:1 with a 36% sucrose solution previously prepared in 100 M Tris-HCL (pH = 9), before being submitted to ultracentrifugation (13,000 rpm/1 h/4 °C). The virus pellet was dissolved in carbonate-bicarbonate buffer (pH 9.6) for the coating of 96-well ELISA plates (ThermoFisher Scientific, Waltham, MA, USA) (100 µL/well), then plates were incubated overnight at 4 °C. On day-2, plates were washed 2x with washing buffer (PBS-Triton X-100, 0.1%) and further blocked with 100 µL/well of 5% skim milk at 4 °C overnight. On day-3, pre-challenge sera were diluted to 1/200 in 5% skim milk, added to wells, and incubated overnight at 4 °C. On day-4, plates were washed 3× before HRP-conjugated anti-chicken IgY antibody (Novus Biologicals, Centennial, CO, USA) was added to wells at a dilution of 1/1000 (100 µL/well) for 1 h at room temperature. After washing 3×, 50 µL of tetramethylbenzidine (TMB) substrate was added to wells for a maximum of 10 min or until color development then 50 µL of 1 M HCL was added to stop the reaction. Plates were scanned at wavelength 450 and values were represented as OD450.

### 2.8. Statistical Analysis

Data was analyzed using GraphPad Prism 9.5.1 software (La Jolla, CA, USA) and displayed as mean and standard deviation (s.d.) of the independent experiments or individual birds (as stated per figure or table legend). The *p*-values were represented as * for *p* ≤ 0.05, ** for *p* ≤ 0.01, *** for *p* ≤ 0.001, and **** for *p* ≤ 0.0001. Data sets included in the analysis passed at least one normality test in GraphPad Prism. The physicochemical characteristics, including intensity PSD sizes, PDI, zeta potential, and encapsulation efficiency) were compared between all three QTAP-pDNA-LNPs formulations using one-way ANOVA followed by Tukey’s Multiple Comparisons test. The log qRT-PCR virus titers and ELISA OD450 reads were analyzed by using one-way ANOVA followed by Dunnett’s Multiple Comparisons test to compare different groups to the control (PBS) one.

### 2.9. Ethics Statement

Chickens were cared for in accordance with established guidelines according to the experimental protocol approved by the UW-Madison Institutional Animal Care and Use Committee (UW-Madison IACUC).

## 3. Results

### 3.1. Construction of IBV-S and IBV-N Plasmids

**Figure 1:**
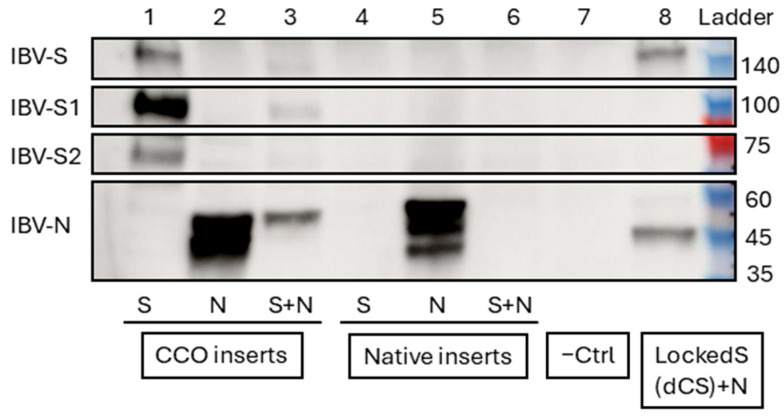
The expression of chicken-codon-optimized (cco) and native IBV-S and IBV-N pDNA. Cells were transfected with a total of 1 µg DNA using Mirus reagent for 3 days before being collected, processed, and submitted for Western blotting. Anti-IBV and anti-chicken IgY antibodies were used for membrane development. Lines 1–3 are cco inserts; Line 1: IBV-S, Line 2: IBV-N, and Line 3: IBV-S + N. Lines 4–6 are native inserts; Line 4: IBV-S, Line 5: IBV-N, and Line 6: IBV-S + N. Line 7: Negative control (−Ctrl.). Line 8: IBV-S + N on same plasmid with a locked IBV-S (deleted cleavage site; dCS) and codon optimization.

To enhance the expression of IBV proteins in chicken, the DNA insert sequences were chicken-codon-optimized (cco) [40], before cloning into the pCAG plasmid. As expected, the cco plasmids successfully expressed the IBV-S or the IBV-N, when transfected into cells (Figure 1). The full IBV-S protein was ~200 KDa in size, which was processed post-translationally into S1 and S2 subunits (~70–100 KDa). The IBV-N protein was ~45–50 kDa in size [4]. Upon transfection of a single pDNA (IBV-S or IBV-N) into cells, the expression of IBV-S protein tentatively increased in cco constructs, compared to native ones. However, the IBV-N protein expression was relatively the same in cco and native inserts (Figure 1). When both IBV-S and IBV-N were equally transfected into cells, (i) the expression of locked full IBV-S (with dCS) was more detectable, compared to the unlocked IBV-S and (ii) the cco IBV-S + N pDNA constructs showed a better protein expression, unlike native inserts (Figure 1). Taken together, cco optimization of pDNA inserts boosted the expression of IBV protein in cell culture.

### 3.2. Formulation and Characterization of QTAP-DNA-LNPs

In Formula I, when DOTAP lipid was dissolved in a 5% glucose solution, the resulting LNPs had a smaller particle size (~215 nm) than other Formulas, but had a high PDI (~0.5), and encapsulated only 35% of pDNA. In Formula II, when DOTAP was dissolved in EtOH, the LNPs had a slightly larger particle size (~233 nm), a lower PDI (~0.33), and higher encapsulation efficiency (~91%) (Table 1). In Formula III, using a higher concentration of DOTAP, the LNP sizes were the largest (~291 nm) and their encapsulation was the highest (~99%) in association with increased DOTAP concentration. However, the PDI values were not largely changed (~0.27), compared to Formula II (Table 1). For zeta potential, Formula I had a negative surface charge (~−22 mV), while Formulas II and III had a positive one (~+21 to +35 mV) (Table 1). Finally, when formulas (I, II, or III) were checked for their intensity PSD, sizes measured in both dispersants (Milli-Q water or 1 mM sodium chloride) matched closely, confirming their colloidal stability.

**Figure 2:**
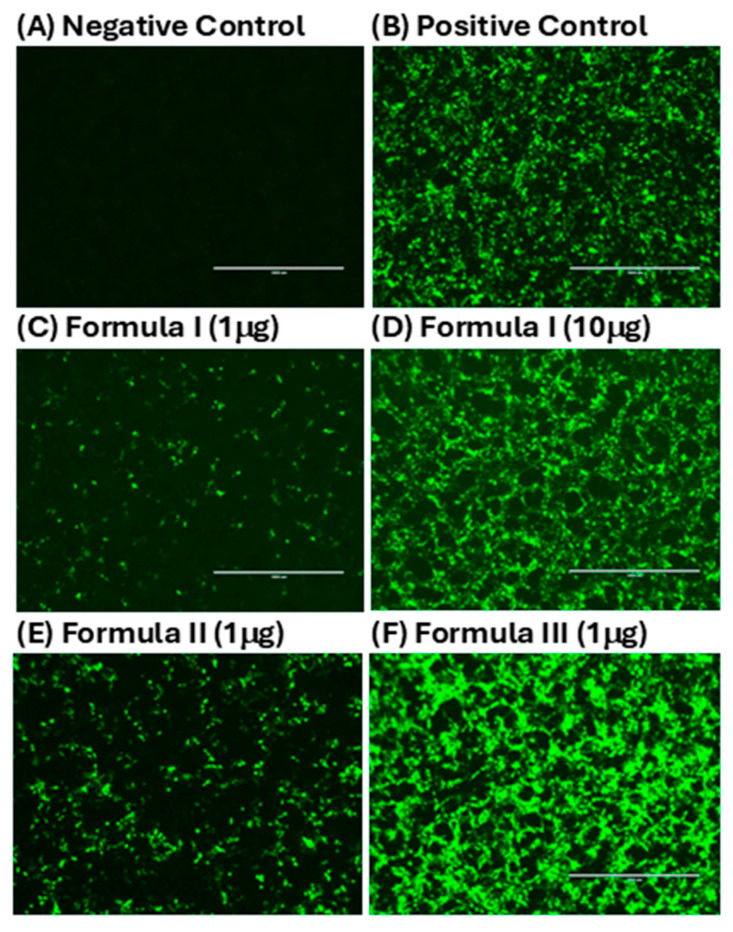
The transfection of QTAP-pDNA-LNPs encoding GFP into HEK293T cells for 3 days. (**A**) Negative control is non-transfected cells. (**B**) Positive control is cells transfected with 1 µg DNA using Mirus reagent MIR-2300. (**C**–**F**) QTAP-pDNA-LNPs with GFP. (**C**,**D**) Formula I was used to transfect (**C**) 1 µg or (**D**) 10 µg DNA. (**E**) Formula II and (**F**) Formula III were used to transfect 1 µg pDNA. Scale bar represents 1000 µm.

**Figure 3:**
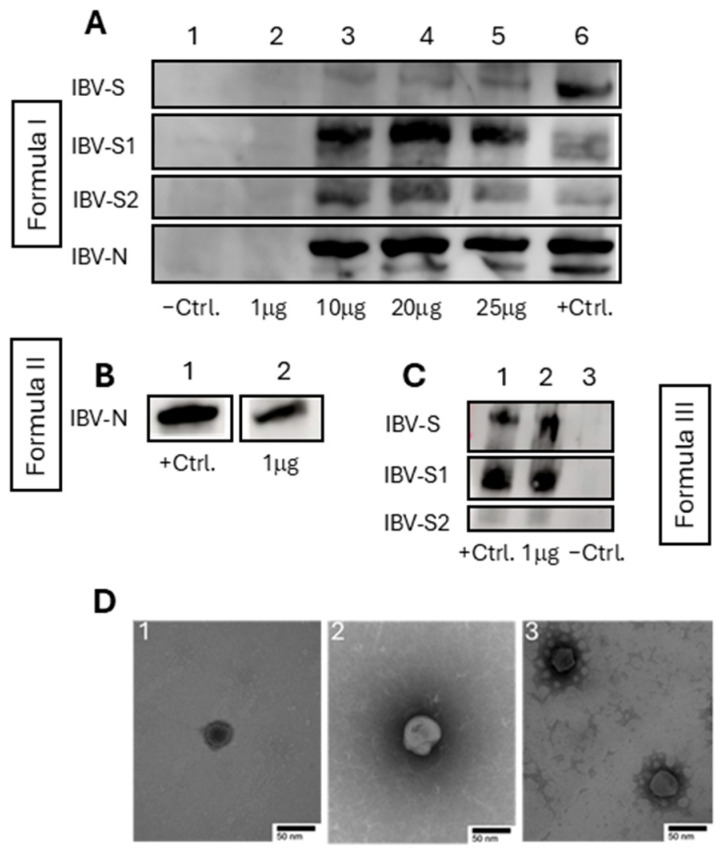
Western blot from cell lysates after transfection of formulated QTAP-pDNA-LNPs. The pDNA (IBV-S or IBV-N or both) was transfected into HEK293T cells for 3 days. Negative control cells (−Ctrl.) are non-transfected, while positive control ones (+Ctrl.) are transfected with 1 µg DNA using Mirus reagent MIR-2300. (**A**) Formula I was used to transfect 1 µg, 10 µg, 20 µg, and 25 µg of IBV-S and IBV-N pDNA (lines 2–5, respectively). Line 1 is the negative control while line 6 is the positive control. (**B**) Formula II was used to transfect 1 µg of IBV-N pDNA. Line 1 is the positive control cells, line 2 is Formula II. (**C**) Formula III was used to transfect 1 µg of IBV-S pDNA. Line 1 is positive control, line 2 is Formula III, and line 3 is negative control. (**D**) Representative transmission electron microscopy (TEM) fields for QTAP-pDNA-LNPs Formulas I (**1**), II (**2**), and III (**3**). Formulas were freshly prepared, then mixed with negative staining and further visualized at a 50 nm scale bar.

To determine the transfection efficiency of the three Formulas, the LNPs encapsulating various concentrations of pDNA (e.g., GFP or IBV genes) were used to transfect HEK293T cells (Figure 2 and Figure 3) and were compared on day-3 post-transfection. Upon transfection of QTAP-pDNA-LNPs Formula I potentially encapsulating 1 µg or 10 µg, GFP expression was positively correlated with the amount of encapsulated DNA (Figure 2C and Figure 2D, respectively). Meanwhile, GFP expression was higher upon using Formula III containing 2.5 mg/mL DOTAP followed by Formula II containing 1.25 mg/mL DOTAP, when 1 µg of GFP-DNA was used (Figure 2E,F). Similarly, the expression of IBV-S and IBV-N proteins was only detectable at high DNA concentrations (10 µg, 20 µg, and 25 µg) but not 1 µg, when Formula I was used (Figure 3A). Meanwhile, both IBV-S and IBV-N were efficiently expressed at 1 µg pDNA concentration using either Formula II or III (Figure 3B,C). By using EM, LNPs appeared spherical and their size varied between 45.4 and 93.5 nm for Formula I, 101.9–253 nm for Formula II, and 72.8–376.3 nm for Formula III (Figure 3D). Other representative TEM fields are also included in Figure S1.

### 3.3. Stability of QTAP-pDNA-LNPs as Vaccine Doses

**Figure 4:**
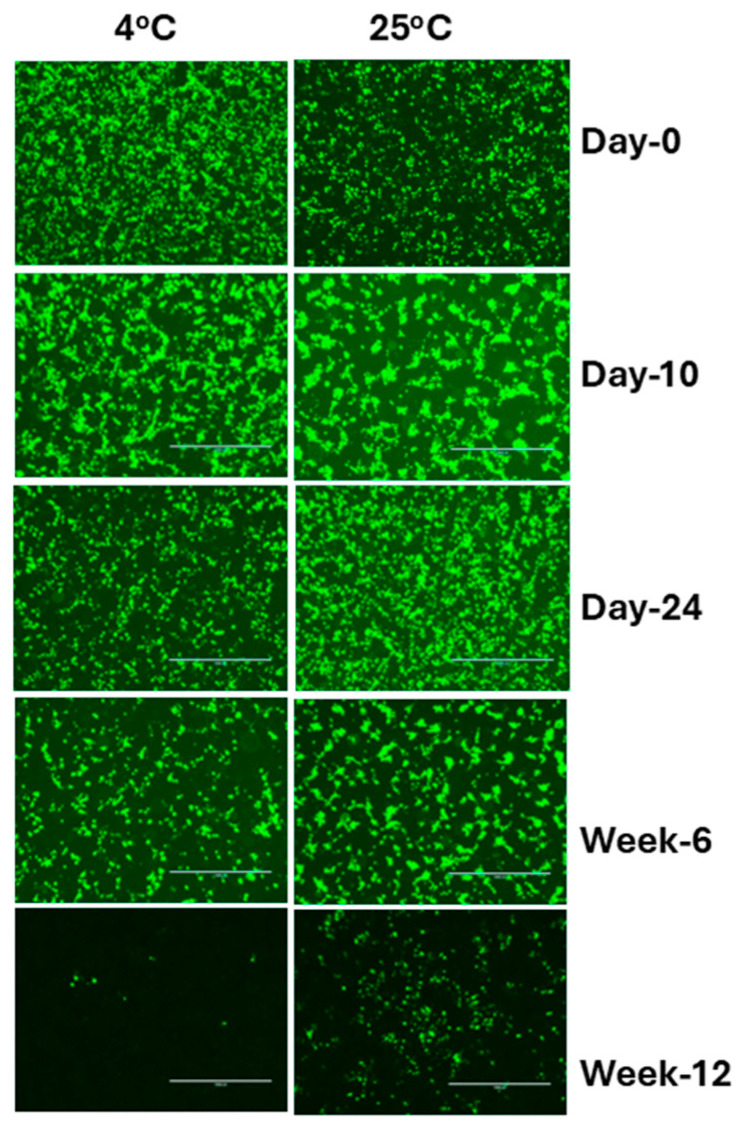
The stability of QTAP-pDNA-LNPs Formula II at indicated timepoints post-formulation. Formula II was prepared on day-0 and kept at either 25 °C or 4 °C for up to 12 weeks. At each timepoint, 1 µg pDNA (GFP) was transfected into cells. Scale bar represents 1000 µm.

The stability of QTAP-pDNA-LNPs Formula II was studied over time at 25 °C (room temperature) and 4 °C (refrigerator temperature) conditions. At each examined point, QTAP-pDNA-LNPs (GFP) were transfected into HEK293T cells at 1 µg concentration. Meanwhile, Formulas were centrifugated at maximum speed, and supernatants were collected to measure the level of unencapsulated DNA. At both conditions (25 °C and 4 °C), Formula II stably expressed GFP in cells up to 6-week post-formulation but showed a tentatively lower GFP expression at 12-week post-formulation (Figure 4). This formula showed a relatively consistent encapsulation efficiency (96.91–99.40%) of pDNA throughout the timepoints of the experiment. When the intensity PSD size and PDI of Formula II were compared, it was revealed that the formula’s size changed from 295.5 ± 22.1 on day-0 to 208.7 ± 35.4 (at 4 °C) or 222.1 ± 24.8 (at 25 °C) at week-12 post-formulation. The formula’s PDI changed only from 0.36 ± 0.005 on day-0 to 0.43 ± 0.078 (at 4 °C) or 0.46 ± 0.037 (at 25 °C) at week-12 post-formulation.

Taken together, we demonstrated that QTAP-pDNA-LNPs vaccine formulas can deliver pDNA vaccine constructs and allow their expression to proteins inside cells to variable degrees. Formulas II and III outperformed Formula I in their characteristics, particularly PDI and encapsulation efficiency. Formula II was also stable at two different storage temperatures, for at least up to 6 weeks post-formulation. Considering the intensity PSD, Formula I had the smallest particle size.

### 3.4. Testing Selected QTAP-pDNA-LNPs Against IBV-M41 in Chicken Model

**Figure 5:**
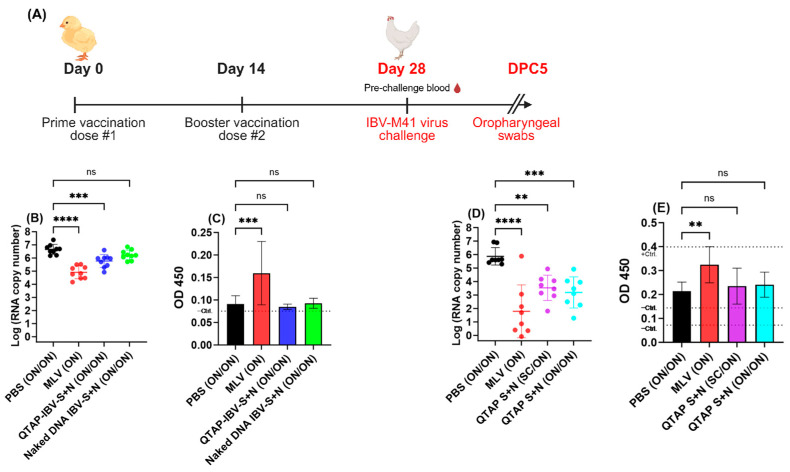
The efficacy of QTAP-pDNA-LNPs against the IBV-M41 challenge in chickens. (**A**) Experimental timeline of both chicken experiments. (**B**,**D**) IBV titers are expressed as log RNA copy number quantified by qRT-PCR. RNA was isolated from swabs collected on day-5 post-IBV challenge (DPC-5) with IBV-M41. (**C**,**E**) Indirect ELISA used to detect anti-IBV IgY levels in pre-challenge sera of chicks at day-28 of age, as values expressed as OD450. Results were compared to the PBS (unvaccinated negative control group). (**B**,**C**) SPF chickens vaccinated with QTAP-pDNA-LNPs Formula I while (**D**,**E**) Commercial chickens vaccinated with QTAP-pDNA-LNPs Formula II. One group received the QTAP-pDNA-LNPs vaccine by SC/ON routes while other groups received it by ON/ON route. Individual values along with mean and s. d. are shown. The one-way ANOVA with Dunnett test was used for statistical multiple comparisons. ** for *p* ≤ 0.01, *** for *p* ≤ 0.001, **** for *p* ≤ 0.0001, and ns means non-significant.

Based on the physicochemical characteristics of formulated QTAP-pDNA-LNPs, we utilized Formulas (I and II) for chicken trials against IBV-M41 in either SPF or commercial chickens, respectively, to delineate the efficacy of QTAP-pDNA-LNPs formulations as a vaccine. Chickens were vaccinated 2 x with QTAP-pDNA-LNPs formulas (encoding IBV-S + N) before IBV challenge (Figure 5A). Comparing overall birds’ health and body weights, no unwanted effects were noticeable among vaccinated and unvaccinated groups for either SPF or commercial chickens (weights ranged from 394.5 ± 414 g to 409 ± 50 in different groups). The SPF chicken group that received QTAP-pDNA-LNPs Formula I showed a statistically significant reduction in tracheal viral shedding, compared to the unvaccinated group (*p* < 0.001). Similarly, statistically significant reductions in tracheal viral shedding were detected in commercial chicken groups that received our QTAP-pDNA-LNPs Formula II by either SC/ON (*p* < 0.01) or ON/ON (*p* < 0.001) routes compared to the unvaccinated group (Figure 5B,D). Likewise, in both SPF and commercial chicken experiments, the MLV-vaccinated birds showed statistically significant reduction in tracheal viral shedding in comparison to the unvaccinated group (*p* < 0.0001). Testing of pre-challenge humoral immunity (systemic IgY) of birds revealed that sera immunoglobin levels against the IBV virus did not change in response to QTAP-pDNA-LNPs vaccination, compared to the unvaccinated group, suggesting that the efficacy of QTAP-pDNA-LNPs vaccine may not be related to IBV-specific humoral immunity. On the contrary, MLV-vaccinated group showed significant elevation in sera IgY levels (Figure 5C,E).

## 4. Discussion

Nucleic acid vaccines encode only the antigen (s) of interest from a pathogen and, by design, avoid the potential risks associated with live-attenuated vaccines, including reversion to virulence, emergence of immune escape mutants, and environmental shedding of seed viruses [10,41,42]. Despite these theoretical advantages, DNA vaccines have not achieved widespread commercial deployment in poultry because of high antigen dose requirements and relatively modest vaccine-induced immunity [43]. In the present work, we leveraged a new form of LNPs as a platform technology to address these limitations and to evaluate two vaccine formulations for protection against IBV challenge in chickens. The QTAP-pDNA-LNPs platform integrates DNA delivery with adjuvant activity by incorporating DOTAP and Quil-A within the formulation to enhance DNA uptake and immunogenicity. For a rational vaccine design against IBV infection, we targeted two IBV structural proteins, S and N antigens. IBV-S contains epitopes that drive receptor binding, immunogenicity, and antigenicity, including neutralizing antibody targets [1]. IBV-N has been reported to stimulate cytotoxic T lymphocyte (CTL) responses [1,44,45]. The full-length IBV-S gene was chosen over S1/S2 subunit constructs due to its conformational dependence, which may preserve epitopes essential for protective immunity [46]. Previously, vaccines against IBV exploited both S and N antigens individually or in combinations, establishing a foundational rationale for our multi-antigen QTAP-pDNA-LNPs system [1,44,47]. To maximize protein expression from plasmid DNA constructs, a Kozak sequence (CCACC) was inserted upstream of the start codon (ATG), the coding sequence was chicken-codon-optimized (cco), the chicken β-actin promoter was used, and LNPs were employed to enhance cellular uptake of DNA [48]. Western blot analysis without loading controls was used to verify the successful protein expression from each plasmid construct. Sequence optimization to codon usage in chickens increased IBV-S expression relative to native sequences, and a locked IBV-S construct demonstrated enhanced expression when paired with IBV-N. We hypothesized that Quil-A-containing adjuvant components in LNPs will optimize LNP characteristics, including size, transfection efficiency, and immunogenicity when used to immunize animals [36,49,50].

The physicochemical properties of the formulated QTAP-pDNA-LNPs, including size, PDI, zeta potential, encapsulation efficiency, and others, are known to influence antigen uptake and hence potential vaccine efficacy [51,52]. Smaller, more uniform particles generally show improved stability and cellular uptake [53], whereas high encapsulation efficiencies are not usually associated with better transfection outcomes [54]. In this study, Formulas I and II approximated ~200 nm in intensity PSD size [55], with TEM confirming the smallest size for Formula I. Meanwhile, Formulas II and III exhibited lower PDI values (~0.2–0.4) and encapsulated > 90% of pDNA, whereas Formula I showed lower encapsulation (~35.5%) and a negative zeta potential, potentially accounting for attenuated transfection efficiency [56] relative to the positively charged, more uniform formulations II and III. Based on those *in vitro* characteristics, we focused our *in vivo* studies on Formula II with high encapsulation efficiency, compared to Formula I with low encapsulation efficiency. However, Formula III was excluded from the *in vivo* testing due to concerns regarding the lipid’s potential cationic toxicity (zeta potential of ~+35) [57] and relatively larger size, which are associated mainly with higher DOTAP concentration used in Formula III, compared to Formula II. Positively charged large LNPs rapidly tend to aggregate in physiological body fluids [56]. As expected from our earlier analysis of QuilA-based vaccines [11,36,58], no undesirable effects were noticed on immunized birds, suggesting the overall safety of the utilized constructs and formulas. More safety analyses could be incorporated in further vaccine efficacy studies using this platform.

The IBV vaccine platform employed here was designed for a prime-boost regimen, given the high infectivity of IBV and the goal of maximizing vaccine-induced immunity [46,59]. In contrast to systemic IgY responses observed with traditional MLV vaccines, the QTAP-pDNA-LNPs did not elicit detectable systemic IgY after vaccination of 1-day-old chicks, highlighting the likelihood that IBV-S/IBV-N antigens produced intracellularly and retained cell-associated expression limits the eliciting of serum antibodies in some contexts [59]. Nevertheless, the LNP-immunized groups demonstrated reduced virus shedding after challenge, a critical functional correlate of vaccine efficacy in IBV models and an indicator of effective potential beyond mere serology. Comparable reductions in shedding have been observed, when Quil-A-adjuvanted DNA vaccines incorporating IBV-N constructs were previously utilized by our group, [11], potentially supporting the likely relevance of Quil-A–driven mucosal and/or cellular immunity. Despite the absence of direct comparison in our study, Formula II generally yielded superior vaccine performance relative to Formula I, consistent with its higher encapsulation efficiency, positive zeta potential, and favorable PDI, which together likely enhanced DNA delivery and immunogenicity. While the small sample size (8–9 birds per group) in this study limits the statistical precision; we acknowledge that smaller cohorts can artificially inflate effect sizes when significance is achieved. Larger high-powered studies are necessary to confirm the scale of this biological response. Notably, Formula II’s stability at room and refrigeration temperatures supports its suitability for field deployment, addressing a known hurdle for DNA-LNP vaccines. Regarding immune correlates and mucosal considerations, IBV protection is driven by both humoral and cellular components, with CTL responses and mucosal immunity playing pivotal roles in controlling respiratory IBV infection. While systemic IgY responses may not always correlate with protection against IBV [59], reductions in viral shedding serve as a practical and meaningful endpoint in vaccine efficacy studies and align with prior observations for Quil-A-adjuvanted DNA constructs and ISCOM-based formulations [60]. In relation to IBV, mucosal immunity is a critical barrier that prevents IBV from primarily infecting the respiratory tract of birds. In addition, cellular responses actively clear the virus and/or virus-infected cells. However, humoral immunity is beneficial in preventing viremia but highly limited over mucosal surfaces [4]. The reductions in viral shedding in vaccinated groups that received QTAP-pDNA-LNPs support that the platform may engage mucosal (IgA) or cellular (T cell) immunity, consistent with previous observations for IBV N-based DNA vaccines and Quil-A–containing adjuvant systems in chickens [11]. Unfortunately, this aspect of the developed vaccine was not fully studied. The exact underlying mechanism of protection of QTAP-DNA-LNPs is to be determined.

Although Quil-A and related saponin adjuvants stimulate both humoral and cellular immunity, their systemic toxicity and hemolytic potential have tempered enthusiasm for their universal adoption, particularly in human vaccines [61]. In veterinary vaccines, Quil-A remains widely used, but translating the adjuvant effects to different species and administration routes requires careful safety profiling and dose optimization [61,62]. Some studies report limited or no systemic antibody induction with certain DNA vaccine formats in very young birds, emphasizing the importance of selecting antigen localization, delivery format, and adjuvant configuration to elicit mucosal and cellular responses in poultry against IBV [11,59]. Additionally, while reductions in viral shedding are an efficacy readout indicator, they do not always coincide with measurable systemic antibody responses, underscoring the need for multi-modal immune readouts (mucosal antibodies and T-cell assays) [11]. In future experiments, exploring alternative saponin-based adjuvants with lower toxicity than Quil-A (e.g., QB-90/QB-80 or licorice-derived saponins like Glabilox variants) may retain adjuvant potency while reducing adverse effects [63,64], which could improve safety profiles for field use. In summary, the QTAP-pDNA-LNPs platform represents a promising DNA vaccine strategy for IBV in poultry, with evidence from the present study indicating that carefully formulated LNPs containing properly selected antigens (IBV-S and IBV-N) and the nanoadjuvant system (DOTAP and Quil-A) can achieve meaningful reductions in viral shedding and efficacy in challenge models. The observed formulation-dependent differences in size, charge, encapsulation, and transfection efficiency provide a mechanistic basis for optimizing vaccine performance. Continued exploration of multi-lipid formulations [65], co-expression strategies (e.g., single plasmid expressing both IBV-S and IBV-N) [66], intracellular antigen targeting [67], and adjunctive cytokines [68] or alternative saponin adjuvants holds promise for advancing DNA vaccine platforms toward practical field deployment in poultry and potentially other species.

## 5. Conclusions

This study reports the development and characterization of different formulations of a DNA vaccine (namely, QTAP-pDNA-LNPs) against IBV. With double immunization, this DNA vaccine can reduce the IBV shedding from IBV-challenged birds, highlighting the platform’s potential to be applied after future improvements.

## Figures and Tables

**Table 1:** The physicochemical characteristics of formulated QTAP-pDNA-LNPs. Formula I had DOTAP initially dissolved in a 5% glucose solution at a concentration of 0.25 mg/mL. Formulas II and III had DOTAP initially dissolved in EtOH, at final concentrations of either 1.25 mg/mL or 2.5 mg/mL, respectively.

QTAP-pDNA-LNPs ^a^	Size (Intensity PSD; d.nm) ^b^	PDI ^c^	Zeta Potential (mV) ^d^	Encapsulation % ^e^
Formula I	215± 43	0.51 ± 0.16	−22 ± 2	35.5 ± 8.5
Formula II	233 ± 61	0.33 ± 0.05	+21 ± 2	91 ± 9.8
Formula III	291 ± 38	0.27 ± 0.04	+35 ± 1.6	99 ± 1.3

^a^ Values represented are the mean and standard deviation (s.d.) of at least three independent trials, as one-way ANOVA with Tukey’s test was used for statistical multiple comparisons. ^b^ *p*-values are non-significant (ns) among all three formulas. ^c^ *p*-values are ≤0.01 for Formula I in relation to Formula II or Formula III. *p*-value is non-significant (ns) between Formula II and Formula III. ^d^ *p*-values are ≤0.0001 for Formula I in relation to Formula II or Formula III. *p*-value is *p* ≤ 0.001 between Formula II and Formula III. ^e^ *p*-values are ≤0.0001 for Formula I in relation to Formula II or Formula III. *p*-value is non-significant (ns) between Formula II and Formula III.

## Data Availability

All data used in this study is included in the paper.

## Supplementary Materials

The following supporting information can be downloaded at: https://www.mdpi.com/article/10.3390/vaccines14070613/s1, Figure S1: Representative transmission electron microscopy (TEM) fields for QTAP-pDNA-LNPs Formulas I (A), II (B), and III (C). Formulas were freshly prepared, mixed with negative staining, and visualized at a 200 nm scale bar.
