# Supplementary material for: A Novel Effective Nanoadjuvant System for Poultry Vaccines
Source: Vaccines (Basel). 2026 Jul 14;14(7):613. doi: 10.3390/vaccines14070613 (PMC13418975; doi:10.3390/vaccines14070613)
Supplement: Supplementary file 1 [file vaccines-14-00613-s001.zip › vaccines-4389518-supplementary.pdf]

(A) Formula (I): 60.6-110.6 nm

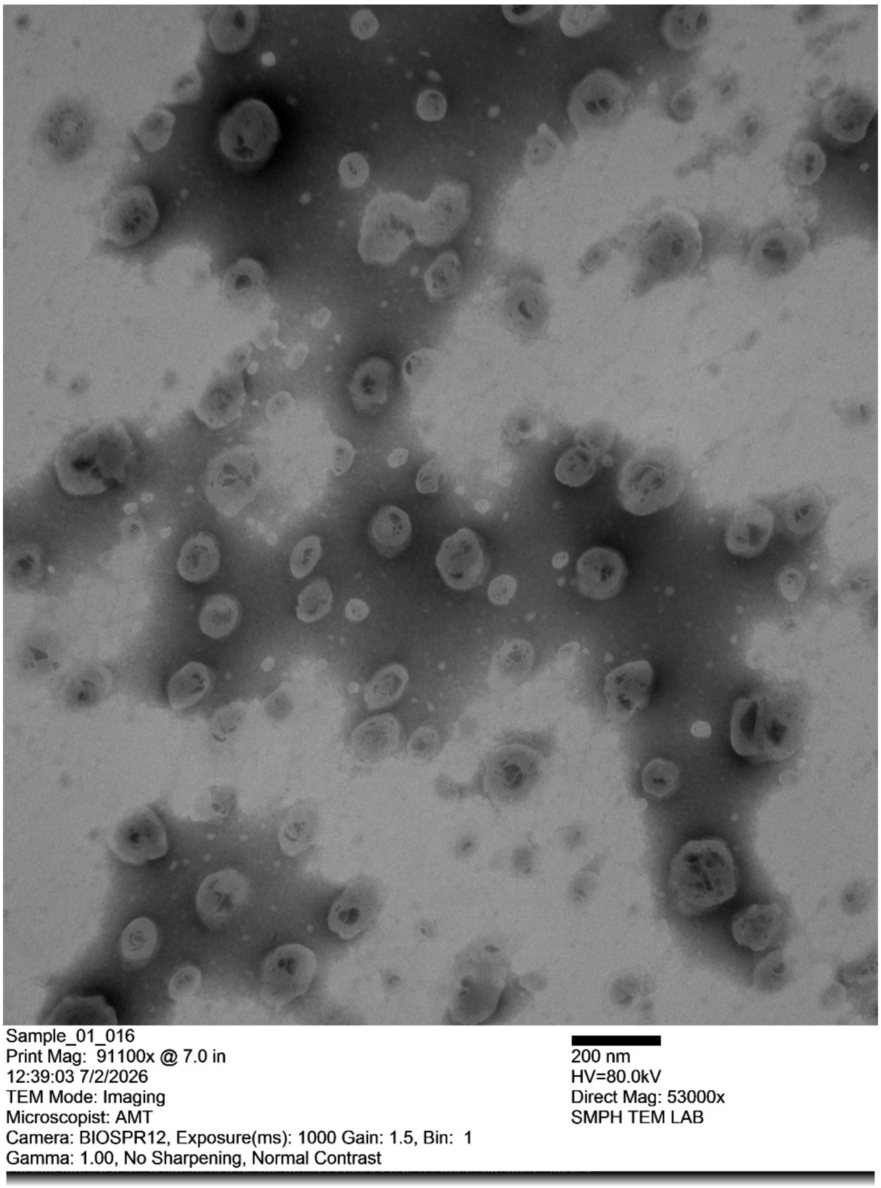

(B) Formula (II): 85.2-191.7

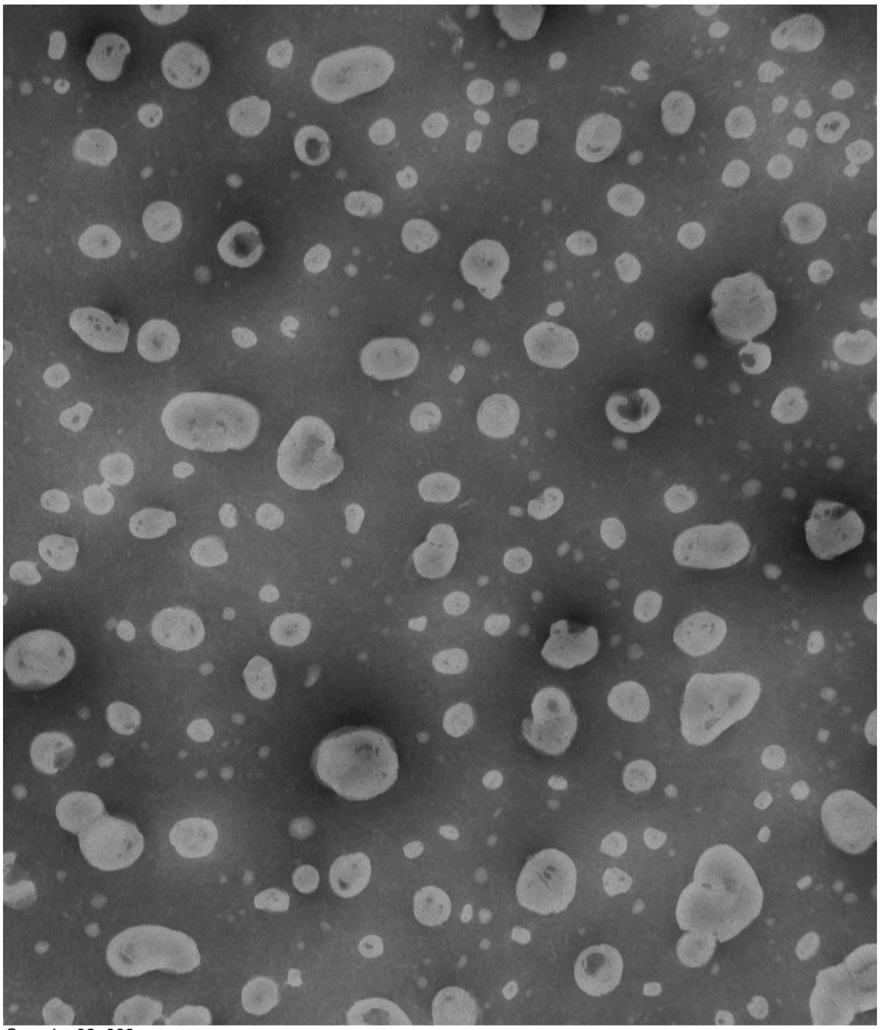

Sample_02_003
Print Mag: 91100x @ 7.0 in
12:50:41 7/2/2026
TEM Mode: Imaging
Microscopist: AMT
Camera: BIOSPR12, Exposure(ms): 1000 Gain: 1.5, Bin: 1
Gamma: 1.00, No Sharpening, Normal Contrast

200 nm
HV=80.0kV
Direct Mag: 53000x
SMPH TEM LAB

(C) Formula (III): 88.7-298.1nm

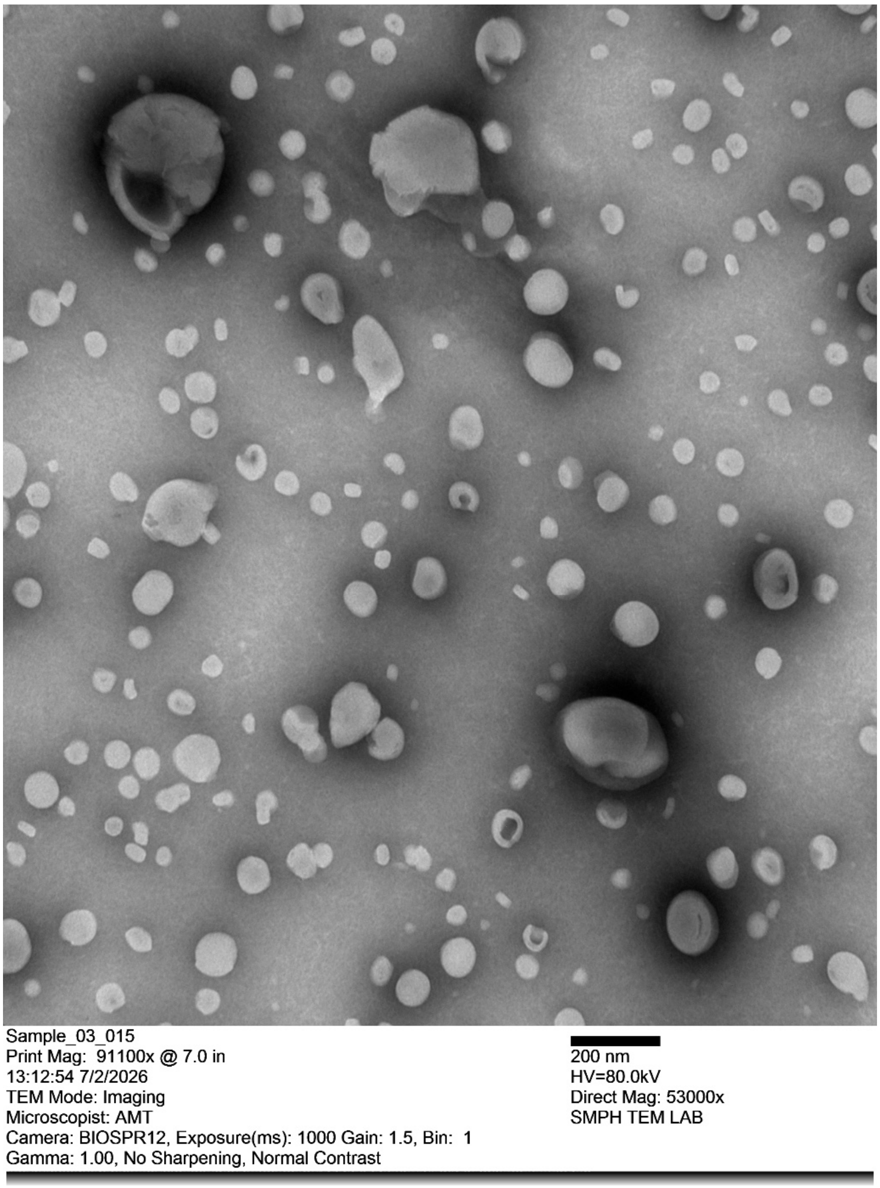

Figure S1. Representative transmission electron microscopy (TEM) fields for QTAP-pDNA-LNPs formulas I (A), II (B), and III (C). Formulas were freshly prepared, mixed with negative staining, and visualized at 200 nm scale bar.
